# In Vitro and In Vivo Anti-Osteoarthritis Effects of 2,3,5,4′-Tetrahydroxystilbene-2-*O*-β-d-Glucoside from *Polygonum Multiflorum*

**DOI:** 10.3390/molecules23030571

**Published:** 2018-03-03

**Authors:** Po-Wei Tsai, Yi-Hui Lee, Lih-Geeng Chen, Chia-Jung Lee, Ching-Chiung Wang

**Affiliations:** 1 School of Pharmacy, College of Pharmacy, Taipei Medical University, Taipei 11031, Taiwan; powei@mail.cjcu.edu.tw; 2 Department of Medical Sciences Industry, College of Health Sciences, Chang Jung Christian University, Tainan 71101, Taiwan; 3 Innovative Research Center of Medicine, Chang Jung Christian University, Tainan 71101, Taiwan; 4 Department of Traditional Chinese Medicine, Wan Fang Hospital, Taipei 11696, Taiwan; b8503052@yahoo.com.tw; 5 Department of Microbiology, Immunology and Biopharmaceuticals, College of Life Sciences, National Chiayi University, Chiayi 60004, Taiwan; lgchen@mail.ncyu.edu.tw; 6 PhD Program for Clinical Drug Discovery of Chinese Herbal Medicine, College of Pharmacy, Taipei Medical University, Taipei 11031, Taiwan; cjlee@tmu.edu.tw; 7 Graduate Institute of Pharmacognosy Science, School of Pharmacy, College of Pharmacy, Taipei Medical University, Taipei 11031, Taiwan; 8 Orthopedics Research Center, Taipei Medical University Hospital, Taipei 11031, Taiwan

**Keywords:** *Polygonum multiflorum*, 2,3,5,4′-tetrahydroxystilbene-2-*O*-β-d-glucoside, osteoarthritis, anti-inflammation

## Abstract

*Polygonum multiflorum* Thunb. is a traditional herbal medicine that is rich in polyphenols. The major compound, 2,3,5,4′-tetrahydroxystilbene-2-*O*-β-d-glucoside (THSG) has many pharmacological activities, such as antioxidative and free radical-scavenging properties, and the abilities to reduce hyperlipidemia, prevent lipid peroxidation, and protect the cardiovascular system. In this study, the anti-osteoarthritis (OA) effects of THSG were explored using in vitro and in vivo models. THSG inhibited nitric oxide (NO) and prostaglandin E_2_ (PGE_2_) production and inducible NO synthase (iNOS) and cyclooxygenase-2 expressions by lipopolysaccharide-stimulated RAW 264.7 cells. On the other hand, THSG inhibited PGE_2_ production and iNOS and matrix metalloproteinase-13 expressions by interleukin-1β-stimulated primary rat chondrocytes. Through a mono-iodoacetate-induced rat OA model assay, THSG reduced paw edema and improved the weight-bearing distribution. Therefore, THSG has anti-inflammatory activity and could be applied as a lead compound for the development as an OA drug.

## 1. Introduction

Osteoarthritis (OA) is a chronic degenerative bone and joint disease mostly in the elderly, with main pathological features of articular cartilage degeneration, mild synovitis, meniscus injury, bone remodeling, subchondral bone sclerosis, and osteophyte formation [1,2,3]. The etiology and detailed pathological mechanisms of OA are still not entirely clear, but age, mechanical wear, genetics, obesity, sex hormones, and other factors are likely to participate [4]. In the past, it was thought that articular cartilage was the main cause of OA degradation, and it has now been confirmed that an imbalance in the destruction and repair processes of articular cartilage leads to OA. The major pathogenic factors of articular cartilage degenerative lesions are joint load conduction disorder, joint nutrition reduction, and free radical damage. Specially, stress will induce reactive oxygen species (ROS), and then ROS causes chondrocyte death by abnormal expressions of cytokines or stimulates the autoimmune abnormalities cause inflammation [4]. All kinds of pathogenic factors can lead to cartilage degeneration, which is caused by different routes of articular chondrocytes and cartilage matrix degradation of the body, in which the death of chondrocytes is the key to cartilage lesions [5,6].

Inflammation is one of the non-specific immune response in the bodies; it normally occurs in any type of injury of the body, which is including harmful irritation, damage to tissues, as well as the production of pathogens, specific disease conditions, and harmful chemicals [7]. According to those above pathogens, inflammation can be divided into acute and chronic stages. Generally, OA is an aging disease, and is not considered to be a kind of inflammatory disease. It is an acute phase of inflammation that is superimposed with crystal or cartilage fragments. However, cartilage OA is classical and inflammatory, and produces prostaglandins (PGs), nitric oxide (NO), and site-related mediators and cytokines, such as interleukin (IL)-1 and tumor necrosis factor (TNF) [8]. On the other hand, accumulating evidence indicates that IL-1β causes chondrocytes to release several proteolytic enzymes that include matrix metalloproteinases (MMPs) and other inflammatory mediators, such as NO and PGE_2_ [9]. Inflammatory mediator levels in synovial fluid and cartilage tissue were found to be elevated in research on OA patients. Thus, inhibiting these inflammatory mediators may diminish the progression of OA [10].

The root of *Polygonum multiflorum* Thunb. (PM) is a traditional Chinese medicine that has been used to invigorate the liver and kidneys, and benefit the essence and blood since the Tang Dynasty more than 1000 years ago. The pharmacological activities of PM have been reported and include antiaging, anticancer, antioxidant, improving immune function, learning, and memory, and hepato-protection [11]. 2,3,5,4′-Tetrahydroxy-stilbene-2-*O*-β-d-glucoside (THSG), a polyhydroxystilbene isolated from the root of PM, is the major constituent of PM. Pharmacological studies indicated that THSG shows potential antioxidation and free radical-scavenging properties; it also reduces hyperlipidemia, prevents lipid peroxidation, and protects the cardiovascular system [12]. Recent research indicated that THSG has the ability to reduce peroxidation levels of the brain in mice with Alzheimer’s disease, decrease the PGE_2_ production, and inhibit cyclooxygenase (COX)-2 expressions in RAW 264.7 cells, and scavenge ROMs [13,14]. However, there are no reports about THSG’s application for arthritis.

In this study, the anti-inflammatory effects of THSG were measured in lipopolysaccharide (LPS)-stimulated RAW 264.7 macrophages and IL-1β stimulated primary cultured rat chondrocytes (PRCs) using in vitro models. In addition, the anti-arthritis effects of THSG were evaluated using a MIA-induced rat OA model. Mono-iodoacetate (MIA) is a glycolytic inhibitor, inhibits glycolysis and induces chondrocyte death in vitro. Intro-articular injection of MIA induces chondrocyte death and causes behavioral, histological, and biochemical change that resemble human’s OA [15]. There are biphasic pain behaviors in the MIA-induced rat OA model. The early phase belongs to an inflammatory phase, and the injected local tissue will be swelling. The late phase is a pain phase after edema eliminating. Therefore, we could use paw edema volume and hind-limb weight bearing distribution as an indirect measurement of MIA-induced rat pain.

## 2. Results

### 2.1. Establishment of the Isolation Protocol of THSG from PM

**Figure 1:**
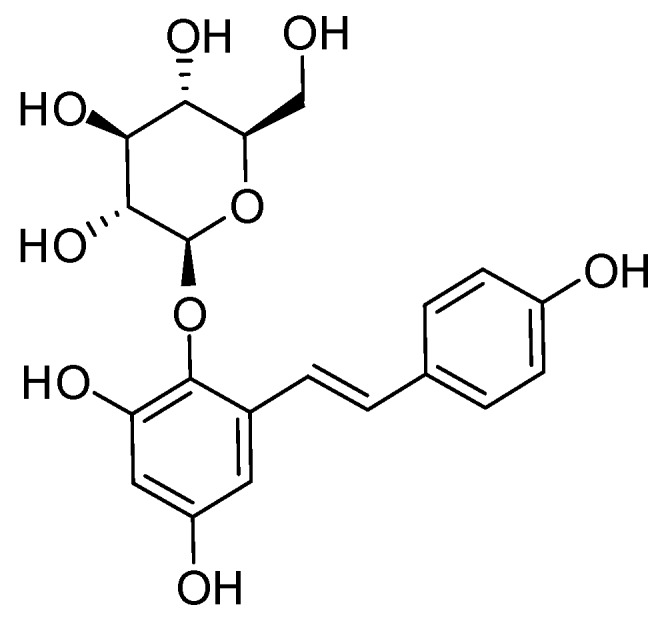
Chemical structure of 2,3,5,4′-Tetrahydroxy-stilbene-2-*O*-β-d-glucoside (THSG).

**Figure 2:**
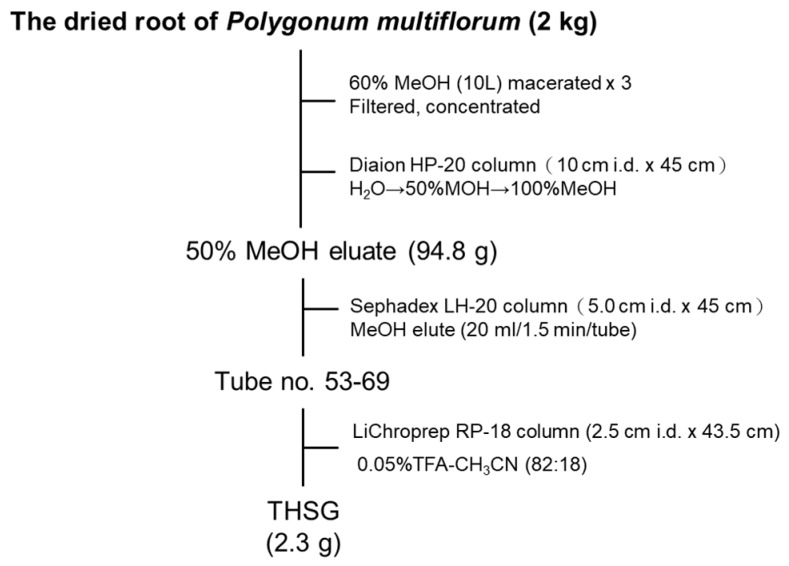
The isolation flowchart of 2,3,5,4′-Tetrahydroxy-stilbene-2-*O*-β-d-glucoside (THSG) from *Polygonum multiflorum* Thunb. (PM).

**Figure 3:**
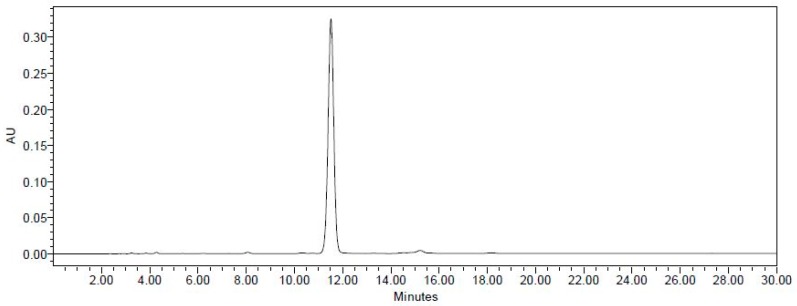
The HPLC chromatogram of 2,3,5,4′-tetrahydroxystilbene-2-*O*-β-d-glucoside. Column: LiChrospher 100 RP-18e (4 mm × 250 mm, 5 μm); Mobile phase: 0.05%TFA-CH_3_CN (83:17); Flow rate: 1.0 mL/min; Column temperature: 40 ℃; Detection: 320 nm.

THSG (Figure 1) is an active compound and enriched in PM; therefore, we established a standard isolation protocol for THSG from PM using Diaion HP-20, Sephadex LH-20, and ODS C_18_ columns (Figure 2). In the isolation system focused on THSG, we excluded other compounds and efficiently gained the active compound, with a 1.2% yield. The purity of THSG was 98.36%, according to a high-performance liquid chromatographic (HPLC) analysis (Figure 3). THSG, ^1^H-NMR (500 MHz, MeOH-d_4_) δ: 7.70 (1H, d, J = 16.5 Hz, olefinic H-a), 7.45 (2H, d, J = 8.6 Hz, H-2′,6′), 6.92 (1H, d, J = 16.5 Hz, olefinic H-b), 6.76 (2H, d, J = 8.6 Hz, H-3′,5′), 6.61 (1H, d, J = 2.8 Hz, H-6), 6.24 (1H, d, J = 2.8 Hz, H-4), 4.50 (1H, d, J = 8.1 Hz, H-1′′), 3.81 (1H, dd, J = 2.5, 11.8 Hz, H-6′′a), 3.76 (1H, dd, J = 4.1, 11.8 Hz, H-6′′b), 3.56 (1H, dd, J = 8.1, 9.0 Hz H-3′′), 3.53 (1H, t, J = 9.0 Hz, H-4′′), 3.43 (1H, t, J = 8.1 Hz, H-2′′), 3.25 (1H, ddd, J = 2.5, 4.1, 9.0 Hz, H-5′′). ^13^C-NMR(125 MHz, MeOH-d_4_) δ: 158.5 (C-5), 156.1 (C-4′) 152.2 ( C-3), 138.1 (C-2), 133.8 ( C-a), 131.0 (C-b), 130.2 ( C-1′), 129.4( C-2′, 6′), 121.9 (C-1), 116.6 (C-3′, 5′), 108.4 (C-6), 103.7 (C-4), 102.8 (C-1′′), 78.4 (C-3′′), 78.1 (C-5′′), 75.6 (C-2′′), 70.9 (C-4′′), 62.2 (C-6′′). The ^1^H and ^13^C NMR data were identical to those previously reported [16].

### 2.2. Anti-Inflammatory Effects of THSG in an In Vitro Assay

**Figure 4:**
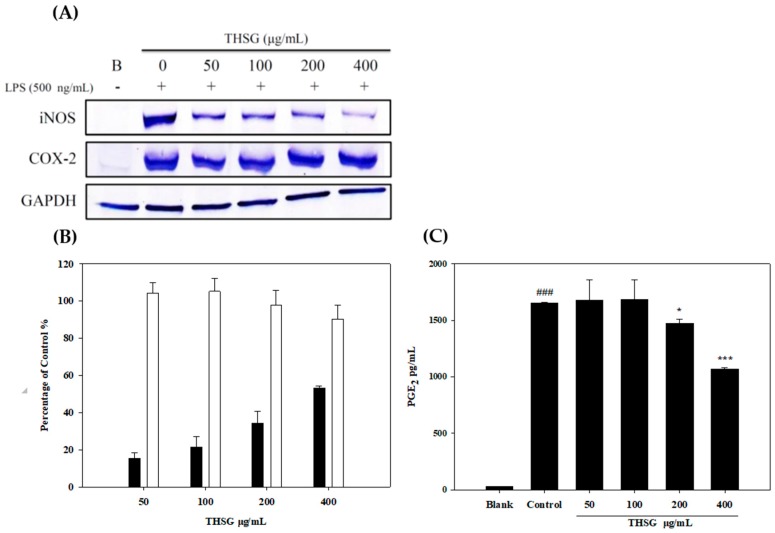
Anti-inflammatory of 2,3,5,4′-Tetrahydroxy-stilbene-2-*O*-β-d-glucoside (THSG) on lipopolysaccharide (LPS)-stimulated RAW 264.7 cells after treatment for 6 h. iNOS and COX-2 protein expressions (**A**); ■ NO production inhibition rate of THSG and ☐ cell viability (**B**); PGE_2_ production level (**C**), #: control group as compared to blank group, ### *p* < 0.005. *: TSHG group compared to control group, * *p* < 0.05, *** *p* < 0.005.

In the inflammatory process, NO and PGE_2_ production are promoted by inducible NO synthase (iNOS) and COX-2, respectively, which are important mediators of inflammatory pain. Using LPS to stimulate mediators of inflammatory reactions and iNOS and COX-2 expressions, NO, and PGE_2_ were produced by RAW 264.7 macrophage cells. Results showed that THSG at 50–400 μg/mL exhibited no cytotoxicity and significantly suppressed iNOS protein expression (Figure 4A) and the NO level in dose-dependent manners (Figure 4B). However, THSG did not significantly downregulate COX-2 expression but significantly decreased the PGE_2_ level (Figure 4C). Therefore, we suggested the PGE_2_ level decrease was through the THSG inhibit the COX-2 activity but not expression.

**Figure 5:**
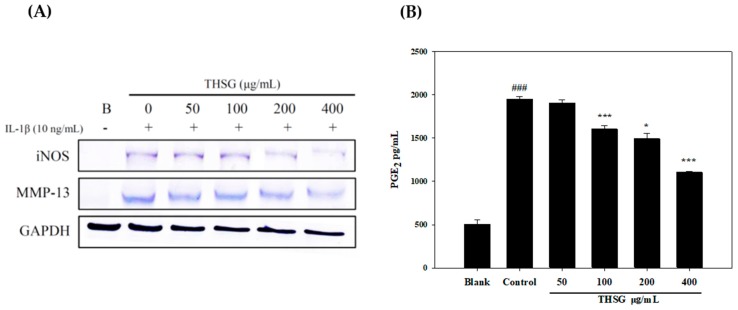
Effects of 2,3,5,4′-Tetrahydroxy-stilbene-2-*O*-β-d-glucoside THSG inhibited iNOS and MMP-13 expression on IL-β induced primary rat chondrocytes (**A**); PGE_2_ production level (**B**), #: control group compared to blank group, ### *p* < 0.005; *: THSG group compared to control group, * *p* < 0.05, *** *p* < 0.005.

The function of MMP-13 is to digest type II collagen of cartilage associated with OA. Therefore, PRCs were isolated from rat cartilage as targeted cells, and we used IL-1β as an inflammatory inducer. In Figure 5, THSG significantly downregulated iNOS and MMP-13 expressions in IL-1β-stimulated PRCs (Figure 5A), and significantly decreased PGE_2_ levels in dose-dependent manners (Figure 5B).

### 2.3. Anti-OA Effects of THSG in an In Vivo Assay

**Figure 6:**
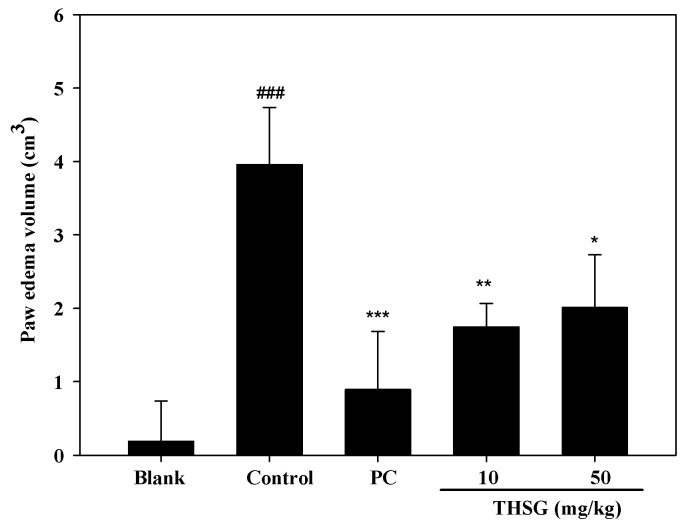
Paw edema volume of rats on mono-iodoacetate (MIA) injection in ankle of rat at 2nd day. Positive control (PC) is indomethacin (2.5 mg/kg). #: control group compared to blank group, ### *p* < 0.005. *: sample group as compared to control group, * *p* < 0.05, ** *p* < 0.001, *** *p* < 0.005.

**Figure 7:**
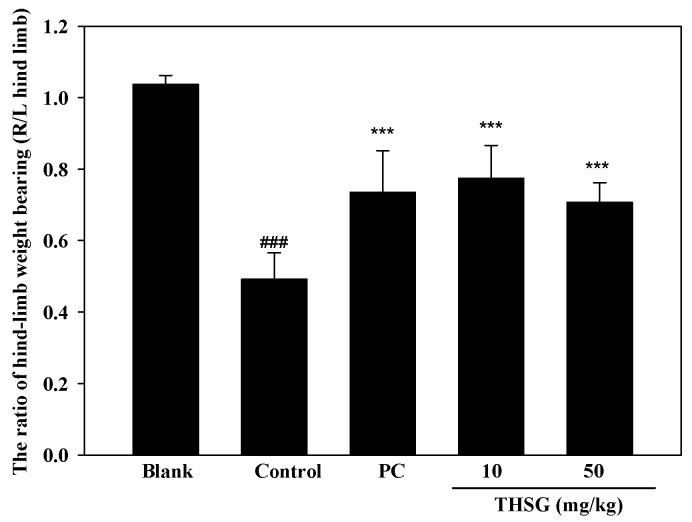
Hind-limb weight bearing ratio of rats on mono-iodoacetate (MIA)-induced osteoarthritis (day 7–day 0). #: control group compared to blank group, ### *p* < 0.005. *: sample group compared to control group, *** *p* < 0.005.

The anti-OA effect of THSG was examined using an MIA-induced OA model in Wistar rats. The model included two stages of arthritis: inflammation and pain. Paw edema was induced by an MIA injection into the right-hind ankle of each rat on day 0. Paw volumes were first measured before the MIA injection on day 0 as the baseline, and then measured a second time on day 2. The increase in the paw volume was calculated by the difference in paw volumes on days 0 and 2. The swelling volume of the paw significantly increased in the control group, and this indicated that an inflammatory response had occurred in the rats. Both THSG and indomethacin markedly reduced paw edema when compared to the control group (Figure 6). Paw edema had recovered by day 6 after the injection, which indicates that inflammation had entered the pain stage. Therefore, an incapacitance tester was used to measure the distribution ratio in hind-limb weight-bearing. The weight-bearing distribution on day 7 in rats was measured with an incapacitance tester for the distribution ratio of hind-limb weight-bearing. In Figure 7, THSG and indomethacin had significantly recovered the hind-limb weight-bearing ratio by day 7. Further, a low dose (10 mg/kg) of THSG exhibited a greater suppressive effect than a high dose (50 mg/kg).

## 3. Discussion

NO and proinflammatory cytokines are produced by macrophages in response to bacterial LPS. The production of NO can be controlled by selective pharmacological inhibition of distinct NOS isoforms [17,18]. iNOS is one of the three key enzymes that generate NO from arginine, and it plays an important role in many body functions. However, the overproduction of NO from macrophages can lead to cytotoxicity, inflammation, and autoimmune disorders, making NO inhibitors essential for preventing inflammatory diseases [19]. PGE_2_ is an important mediator of the inflammatory process and it is produced at inflammatory sites by COX-2 [20]. Both the induction of COX-2 activity and subsequent generation of PGE_2_ are closely related to NO production [21], and so reducing levels of both substances might be an effective strategy for inhibiting inflammation.

IL-1β is one of the inflammatory cytokines that is associated with the degradation of extracellular matrix (ECM) components and plays critical roles in the progression of OA [22]. IL-1β can induce iNOS and COX-2 expressions, leading to the production of NO and PGE_2_. Elevated NO and PGE_2_ levels are very important values to observe in OA patients [23]. NO can induce MMP production and activation, and it also inhibits collagen-type II and proteoglycan synthesis in OA [24]. PGE_2_ can exert many pathological effects that suppress chondrocyte proliferation and inhibit ECM synthesis in the pathogenesis of OA [25]. MMPs are proteases that include various functions. MMP-1 and MMP-13 were shown to play important roles in OA progression. Thus, potential OA therapy has focused on targeting MMPs to degrade the ECM [26]. Furthermore, macrophages are involved in nonspecific inflammation in the articular cartilage. Therefore, LPS stimulated RAW264.7 macrophages and then induced inflammatory response that was a good in vitro screening model for the anti-inflammation effects of natural products. Therefore, in this experimental study, we firstly explore the NO, PGE_2_ production, iNOS and COX-2 expression of THSG in LPS-stimulated RAW 264.7 macrophage. Our results as same as Zhang et al. reported THSG could inhibit COX-2 expression and activity [14]. However, Figure 4 did not show THSG significantly inhibit COX-2 expression, only slightly inhibition. The major different factor was that we did not use RT-PCR enlarge the protein, the sensitivity was too low. On the other hand, we would like to more focus on articular cartilage, therefore using IL-1β-stimulated PRCs as the other anti-inflammatory in vitro model. In IL-1β-stimulated PRCs, THSG also suppressed PGE_2_ production and iNOS and MMP-13 expressions. The MMP-13 is a human collagenase-3 that plays an important role in type II collagen degradation in OA. Type II collagen is the preferred substrate for MMP-13 and its expression and contents of MMP-13 is upregulated in human OA cartilage [27]. According to the above data, we suggest that THSG can inhibit inflammatory responses in joint cavities and the degradation of cartilage.

MIA significantly affects the metabolism of chondrocytes and also causes lower-bone cartilage lesions and chondrocyte death. Pathological changes are very similar to those of OA in humans [15]. It was reported that MIA can lead to qualitative cartilage degradation and apoptosis of chondrocytes [28]. OA is seen as non-inflammatory arthrosis with localized inflammation characterized by cartilage degeneration [29].

Currently, drug treatment is mainly used to alleviate OA swelling, pain, muscle tension, and other symptoms, but these drugs cannot cure or reverse the progression of OA. Therefore, if it is possible to find a drug that can prevent or inhibit the onset of OA progression, it would be an important leap in patient care. Related research reports have shown that THSG has a variety of biological functions, such as anti-inflammatory effects, delaying senescence, cardiovascular protection, neuroprotective effects, amelioration of diabetes, and the promotion of hair growth [30,31,32,33].

In our MIA-induced OA model, THSG reduced paw edema and recovered hind-limb weight-bearing, which indicated that THSG can inhibit the progression of OA. However, THSG at a low dosage (10 mg/kg) had better pharmacological properties than at a high dosage (50 mg/kg). The results showed that the high dosage THSG possibly induced other toxic effects and suppressed the pharmacological effects. In traditional clinical use, processed PM can better protect the knee than raw PM. Therefore, PM must be processed with steam to reduce the THSG content [34].

## 4. Materials and Methods

### 4.1. Plant Materials

Root of P. multiflorum was purchased from a traditional Chinese medicine store in China in 2014. The plants were identified by the Industrial Technology Research Institute in Taiwan. Voucher specimens of dried roots were deposited as 2014 He-Shou-Wu 1 at the Graduate Institute of Pharmacognosy, College of Pharmacy, Taipei Medical University, Taipei, Taiwan.

### 4.2. Extraction and Isolation of THSG

Dried slices of roots of PM (2 kg) were pulverized and soaked in 60% methanol (10 L) for one day. After being filtered, the residues were twice extracted with 60% methanol (10 L). The filtrate was combined and concentrated under a vacuum with a rotary evaporator to obtain an aqueous solution. The aqueous solution was chromatographed on a Diaion HP-20 column (10 cm i.d × 45 cm, Mitsubishi Chemical Industry, Tokyo, Japan) eluted with H_2_O, 50% MeOH, and 100% MeOH (each 5 L). The 50% MeOH eluate (94.8 g) of the Diaion HP-20 column was chromatographed over a Sephadex LH-20 column (5.0 cm i.d. × 45 cm, GE Healthcare Biosciences, Uppsala, Sweden) eluted with MeOH (20 mL/1.5 min/tube) to give a THSG-enriched fraction (tube no. 53-69). The THSG-enriched fraction (5 g) dissolved in water was chromatographed on a LiChroprep RP-18 column (2.5 cm i.d. × 43.5 cm, Merck, Darmstadt, Germany) eluted with 0.05% trifluoroacetic acid–CH_3_CN (82:18) to give an amorphous powder of THSG (2.3 g). The purity of THSG was 98.7% determined by HPLC (Waters 1525 binary pump connected with 717 plus autosampler and 2487 dual λ absorbance detector, Waters, Milford, MA, USA). A flowchart of THSG isolation is shown in Figure 2. The structure of THSG (Figure 1) was identified by comparison of its ^1^H and ^13^C nuclear magnetic resonance (NMR), (Bruker Avance DRX 500MHz, Bruker Corp., Billerica, MA, USA) data with those reported in the literature [16].

### 4.3. Anti-inflammatory In Vitro Assay

#### 4.3.1. Cell Cultures

The RAW 264.7 macrophage cell line was obtained from American Type Culture Collection (Rockville, MD, USA). Cells were maintained in Dulbecco’s modified Eagle’s medium (DMEM) supplemented with 100 U/mL penicillin, 100 streptomycin μg/mL, and 10% fetal bovine serum (FBS) from Gibco BRL (Grand Island, NY, USA), and then incubated at 37 °C in a humidified incubator containing 5% CO_2_.

#### 4.3.2. Primary Chondrocyte Culture

Primary rat chondrocytes (PRCs) were isolated from cartilage tissue of a rat’s knee joint which was then sequentially digested with pronase (10 g/L) (Roche, Basel, Swiss), for 30 min and type IV collagenase (1 g/L) (Sigma, Saint Louis, MO, USA) for 6 h, as described previously [20]. The third passage of cells was used for all of the experiments. Monolayer cultures were established in 60-mm Petri dishes at a concentration of 6 × 10^6^ cells/mL in DMEM supplemented with 10% FBS, 100 mg/L streptomycin, and 100 IU/mL penicillin (Gibco, Waltham, MA, USA). Cell cultures were incubated at 37 °C in a humidified atmosphere of 5% CO_2_.

#### 4.3.3. Measurements of iNOS, COX-2, and MMP-13 Protein Expressions

Methods were performed, as described previously, with some modification [35]. Lipopolysaccharide (500 ng/mL) was used to stimulate RAW 264.7 cells, and IL-1β (10 ng/mL) was used to stimulate PRC cells; then iNOS and COX-2 were overexpressed in RAW 264.7 cells and iNOS and MMP-13 in PRCs, respectively. After co-culture of a sample and inducer for 24 h, cells were harvested by trypsin. Whole-cell lysates from treated cells including a sample were prepared by washing with phosphate-buffered saline (PBS) and lysed with a radioimmunoprecipitation assay (RIPA) buffer. Total protein samples (30 μg) were used for a Western blot analysis, and proteins were transferred to nitrocellulose membranes. Membranes were probed with antibodies specific for iNOS, COX-2, and MMP-13, and then visualized using a BCIP/KBT kit (Gibco, Waltham, MA, USA).

#### 4.3.4. Measurement of NO and PGE_2_ Production

This assay was applied, as previously described [35]. Briefly, cells generated NO and PGE_2_ in the medium after 24 h of incubation with or without samples and/or inducers. NO and PGE_2_ were produced from LPS (500 ng/mL)-stimulated RAW 264.7 cells, and PGE_2_ was also produced from IL-1β (10 ng/mL)-stimulated PRC cells. In addition, culture medium was collected after 24 h of incubation with a sample, and NO and PGE_2_ concentrations were determined. The NO level was measured at 530 nm after the Griess reaction, and PGE_2_ was measured with an enzyme-linked immunosorbent assay (ELISA) kit (Amersham Pharmacia Biotech, Buckinghamshire, UK). The inhibition percentage (%) was calculated by the equation: inhibition (%) = [1 − (T/C)] × 100%, where T and C represent the mean optical densities of inducer stimulated-cells with or without a sample, respectively.

### 4.4. Anti-inflammatory In Vivo Assay

#### 4.4.1. Animals

Male Wistar rats weighing approximately 400–450 g were maintained at 21 ± 2 °C with food and water and kept on a 12-h light/12-h dark cycle. All of the rats used in this experiment were cared for according to the Ethical Regulations on Animal Research of Taipei Medical University (approval no: LAC-100-0043).

#### 4.4.2. MIA-induced OA Model

**Figure 8:**
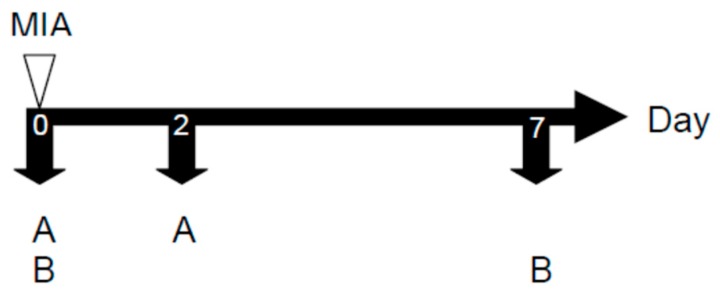
The experimental schedule of mono-iodoacetate (MIA)-induced osteoarthritis model. MIA: first time injected 80 mg/mL MIA, 80 μL. (**A**) Paw edema measurement; (**B**) Hind-limb weighting bearing measurement.

Five groups of eight male Wistar rats each were divided as follows: blank, control group, positive control group (PC), and THSG groups. Male Wistar rats were induced twice by intra-articular injections of 50 μL of 80 mg/mL MIA (Sigma, St. Louis, MO, USA) into the ankle using a 100-μL syringe (Figure 8). Rats were randomly divided into eight animals per group. After the MIA injection, the THSG groups were orally administered 10 or 50 mg/kg THSG daily for seven days. The PC group was treated with 2.5 mg/kg indomethacin daily for 7 days.

The change of the paw volume was measured with a plethysmometer (Ugo Basile, Comerio VA, Italy) on days 0 and 2 after the MIA injection. The weight-bearing of both hind limbs was observed with an incapacitance tester with a dual-channel weight averager (Linton Instrumentation, Norfolk, UK) on days 0 and 7 after the MIA injection [15]. The hind-limb weight-bearing distribution between the right (MIA-induced side) and left limbs was evaluated as an index of joint discomfort in the OA ankle. Weight-bearing as measured in the hind limb was averaged over a 2-s period. Each data point was the mean ± SD by three duplicate readings. The distribution ratio of the right (MIA injection side) and left hind-limb (control side) weight-bearing was assessed by the following equation: mean weight-bearing of right hind-limb/mean weight-bearing of the left hind-limb. The above three kinds of tests were executed after 1–2 h of oral treatment.

### 4.5. Statistical Analysis

Each experiment was performed at least in triplicate. Results are presented as the mean ± standard deviation. Student’s *t*-test was used to analyze data of the animal models.

## 5. Conclusions

Macrophages play an important role in inflammation through several pro-inflammatory mediators’ production. THSG could inhibit NO, PGE_2_ production, iNOS expression, and COX-2 activity in LPS-stimulated RAW 264.7 macrophages. The other hand, chondrocyte activity directly influences to the functions of articular cartilage. Moreover, THSG could inhibit PGE_2_ production and iNOS and MMP-13 expressions in IL-1β-stimulated primary rat chondrocytes. In MIA-induced rat OA model, THSG could reduce paw edema and improve the weight-bearing distribution. The above results provide evidence to support the correlation between OA pathogenesis and the inflammatory response for anti-degenerative arthritis. Therefore, we suggest that THSG could be as a lead compound to develop an OA treatment agent.
